# Occlusion-Aware Face Recognition via Adaptive Local Feature Fusion and Identity-Guided Contrastive Learning

**DOI:** 10.3390/s26133977

**Published:** 2026-06-23

**Authors:** Kexin Zhu, Guoqing Ma

**Affiliations:** 1 School of Mechatronic Engineering, Changchun University of Science and Technology, Changchun 130022, China; 2023100540@mails.cust.edu.cn; 2 Chongqing Research Institute, Changchun University of Science and Technology, Chongqing 401133, China

**Keywords:** partial occlusion facial recognition, occlusion perception, adaptive weighting, local feature fusion, contrastive learning

## Abstract

Partial occlusion can substantially impair the accuracy and stability of face recognition systems. Although existing methods perform well on unobstructed face images, their performance often degrades under partial occlusion, because occluded regions may obscure discriminative identity cues and introduce noise into feature representations. To address this issue, this paper proposes an occlusion-aware face recognition framework that integrates lightweight feature extraction, local-region reliability modeling, adaptive feature fusion, and joint loss optimization. Specifically, the face is divided into three sub-regions according to common occlusion patterns, and an MLP-based module is used to estimate the reliability of each region. The estimated reliability weights are then used to adaptively fuse local features, thereby emphasizing visible and discriminative regions. In addition, a joint loss combining ArcFace and InfoNCE is constructed to enhance inter-class separability and intra-class feature consistency. Experimental results under masks, hats, sunglasses, and random occlusion conditions show that the proposed method achieves a recognition accuracy of 92.3%. Compared with ArcFace, CurricularFace, and AdaFace, the proposed method improves accuracy by 9.9%, 6.5%, and 4.1%, respectively. In addition, the FAR is reduced by 5.8%, 4.9%, and 3.7%, respectively, while the FRR is reduced by 2.2%, 6.5%, and 1.3%, respectively. These results demonstrate that the proposed framework effectively enhances the robustness of face recognition under partial occlusion.

## 1. Introduction

Partial occlusion can substantially impair the accuracy and stability of face recognition systems. Although existing methods perform well on unobstructed face images, their performance often degrades under partial occlusion, because occluded regions may obscure discriminative identity cues and introduce noise into feature representations. To address this issue, this paper proposes an occlusion-aware face recognition framework that integrates lightweight feature extraction, local-region reliability modeling, adaptive feature fusion, and joint loss optimization.

Specifically, the face image is horizontally divided into three sub-regions according to common occlusion patterns. LA-ResNet50 is then used to extract features from each facial region, and an MLP-based module is employed to estimate the reliability of each region. The estimated reliability weights are subsequently used to adaptively fuse local features, thereby emphasizing visible and discriminative regions. In addition, a joint loss combining ArcFace and InfoNCE is constructed to enhance inter-class separability and intra-class feature consistency.

Experimental results under mask, hat, and sunglasses occlusion conditions show that the proposed method achieves a recognition accuracy of 92.3%. Compared with ArcFace, CurricularFace, and AdaFace, the proposed method improves accuracy by 9.9%, 6.5%, and 4.1%, respectively. In addition, the FAR is reduced by 5.8%, 4.9%, and 3.7%, respectively, while the FRR is reduced by 2.2%, 6.5%, and 1.3%, respectively. These results demonstrate that the proposed framework effectively enhances the robustness of face recognition under partial occlusion.

## 2. Related Work

### 2.1. Occluded Face Recognition

Face recognition is a fundamental computer vision task that aims to determine a person’s identity from facial images. With the development of deep learning, deep face recognition methods based on discriminative feature embeddings have achieved significant progress and have become a mainstream solution for identity recognition tasks [1]. Compared with traditional hand-crafted feature methods, deep learning-based approaches show stronger capabilities in nonlinear feature representation, high-dimensional data processing, and discriminative feature extraction. However, most conventional face recognition models are designed for complete or near-frontal facial images. When these models are directly applied to partially occluded facial images, their recognition performance may drop significantly. In practical scenarios, facial images are often affected by masks, sunglasses, hats, hands, and other accessories. These occlusions may cover key identity-related regions and introduce irrelevant visual interference, resulting in the loss of effective identity information and reduced recognition accuracy and stability [2].

Existing studies on occluded face recognition mainly focus on facial feature restoration and attention-enhanced recognition. Facial feature restoration methods attempt to recover missing or corrupted facial regions before recognition, while attention-enhanced methods improve the robustness of feature representation by guiding the model to focus on more discriminative facial regions.

### 2.2. Facial Feature Restoration Methods

Facial feature restoration methods aim to reconstruct missing or corrupted facial regions by generating semantically reasonable and contextually coherent facial details. These methods usually involve facial degradation modeling and deep learning-based image reconstruction [3]. Existing restoration-based methods attempt to reconstruct or recover occluded facial regions using generative facial priors, image inpainting, or de-occlusion strategies [4,5,6]. Some studies further combine facial restoration, identity information preservation, and recognition tasks to improve the identity consistency of restored facial images [7].

Although restoration-based methods can improve the visual completeness of occluded faces, they still have several limitations. First, these methods usually require complex network structures, paired high-quality and low-quality facial images, and accurate identity annotations, which increases the difficulty of data acquisition and model training. Second, the reconstructed facial details may not always correspond to the true identity information of the original face. When large occlusions occur, the generated regions may introduce artificial identity bias, which can further affect the subsequent recognition process. Therefore, when the missing regions contain important identity-related information, restoration-based methods may suffer from insufficient reliability.

### 2.3. Attention-Enhanced Face Recognition Methods

Attention-enhanced face recognition methods introduce attention mechanisms into deep neural networks to guide the model to focus on discriminative and reliable facial regions. Existing studies commonly use channel attention, spatial attention, or self-attention mechanisms in Transformer-based architectures to model global facial structures and local facial details [8,9,10,11,12,13]. These methods can adaptively enhance useful facial features and suppress occlusion interference, thereby improving recognition robustness in complex scenarios [14].

In addition, attention-guided pyramidal feature learning has also been explored in fine-grained image recognition to extract discriminative multi-level features from limited samples. This indicates that attention mechanisms combined with pyramidal feature representation can effectively enhance fine-grained visual recognition. However, such methods are mainly designed for general fine-grained image recognition tasks and do not explicitly model the reliability of local facial regions under occlusion conditions.

Most attention-based methods mainly enhance feature responses from a global or appearance-driven perspective. They usually rely on visual saliency or feature activation strength to assign attention weights, but highly activated regions are not necessarily reliable for identity recognition. For example, occluding objects such as masks, sunglasses, or hats may also produce strong feature responses, causing the model to encode occlusion noise together with valid identity information. In addition, many attention-based methods do not explicitly evaluate the reliability of different local facial regions under different occlusion conditions. As a result, unreliable occluded regions may still contribute to the final feature representation, while discriminative non-occluded regions may not be sufficiently emphasized.

### 2.4. Limitations of Existing Methods

In addition to restoration-based and attention-enhanced methods, some recent studies have attempted to improve face recognition performance by optimizing network architectures and training strategies for feature extraction [15,16,17,18]. Transformer-based recognition frameworks have also been applied to other visual recognition tasks, such as structural damage identification, showing their ability to learn discriminative representations under complex conditions [19,20]. Although these face recognition methods have improved recognition performance to some extent, many of them are still designed based on complete or ideal facial samples.

In summary, existing occluded face recognition methods have improved recognition robustness from different perspectives, such as facial feature restoration, attention enhancement, and feature representation optimization. However, restoration-based methods may introduce identity bias when reconstructing missing facial regions, while attention-based methods usually rely on global or appearance-driven feature responses and lack explicit modeling of local-region reliability. More specifically, the main limitation lies in the lack of an identity-aware criterion for judging whether a local facial region is truly reliable for recognition. Existing methods may assign high responses to visually salient occluders, such as masks or sunglasses, but these regions do not necessarily contain valid identity information. As a result, local regions with strong activation may still introduce occlusion noise into the final representation, while truly discriminative visible regions may not be sufficiently emphasized. In addition, existing methods often struggle to adaptively determine the effectiveness of each local region for identity representation under different occlusion positions and degrees, and have difficulty distinguishing highly activated occluded regions from truly reliable identity-related regions. Although previous studies have explored local feature learning and identity-preserving constraints, how to use identity-consistency constraints to guide local-region reliability modeling and achieve adaptive fusion of reliable local features under different occlusion conditions still requires further investigation. To address these limitations, this study introduces an identity-guided local-region reliability modeling strategy, which adaptively estimates the reliability of different facial regions and fuses reliable local features under partial occlusion.

## 3. Proposed Methods

### 3.1. Overall Framework

**Figure 1:**
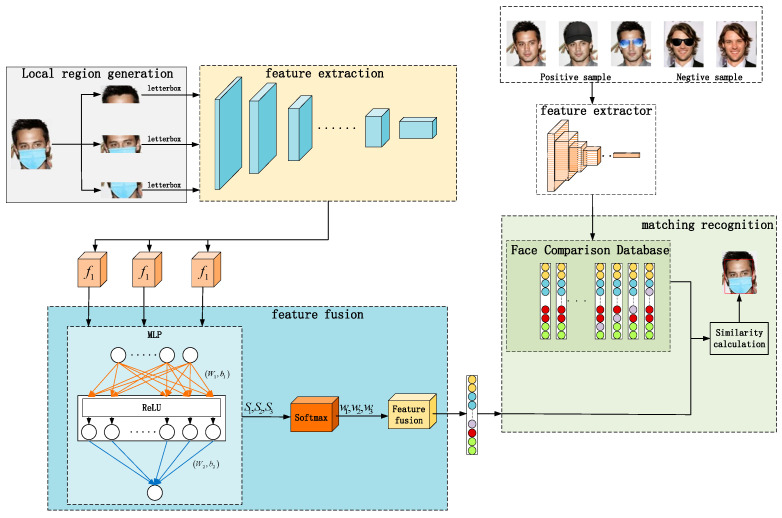
The local occlusion facial recognition framework proposed in this article. The aligned and resized face image is divided along the height dimension into three local image patches, corresponding to the upper, middle, and lower facial regions. The corresponding feature maps, denoted as $f_{1},\;f_{2},\;f_{3}$, are then extracted using a feature extractor. These features are fed into an MLP to pre-dict original contribution scores $S_{1},\;S_{2},\;S_{3}$. A Softmax function then converts these scores $S_{1},\;S_{2},\;S_{3}$ into normalized weights $w_{1},\;w_{2},\;w_{3}$ which are used to adaptively fuse the three local features through weighted summation.

As shown in Figure 1, different colored boxes represent distinct functional modules: yellow indicates the feature extraction network (LA-ResNet50) [21], blue denotes the feature fusion process, and green represents the identity recognition process. The proposed occlusion-aware face recognition framework consists of three core modules: feature extraction, feature fusion, and matching recognition. First, three local image patches are generated by horizontally partitioning the aligned and resized face image: the upper, middle, and lower facial regions. Subsequently, the feature extraction module uses LA-ResNet50 to separately extract the identity features of each local region. Next, the feature fusion module predicts the original contribution score of each local region through a two-layer MLP and uses Softmax to generate normalized weights, thereby achieving adaptive weighted fusion of local features. Finally, the fused feature is compared with the facial feature vectors in the database through similarity calculation to complete identity authentication. The following subsections provide a detailed description of each component of the framework.

### 3.2. Local Facial Region Generation

Given a resized face image $I\in R^{H\times W\times C}$, local facial regions are generated by uniformly dividing the image along the height dimension. Specifically, the image is divided into three horizontal local patches, denoted as $p_{1}$, $p_{2}$, and $p_{3}$, corresponding to the upper, middle, and lower facial regions, respectively. Let h=[H/3]. The first two patches have a height of h, while the last patch covers the remaining pixels to preserve the complete image region. The three patches are defined as

(1) $$p_{1}=[0:h,:,:],\;\;p_{2}=[h:2h,:,:],\;\;p_{3}=[2h:H,:,:],$$

where $h=\left[H/3\right]$ patches are then used as the inputs to the subsequent feature extraction network.

### 3.3. Lightweight Feature Extraction with L-BTNK1

To reduce computational complexity while preserving discriminative facial representations, we construct a lightweight feature extraction network named LA-ResNet50 based on the original ResNet50. Compared with ResNet50, LA-ResNet50 retains the stage-wise backbone architecture but introduces two main modifications. First, the original BTNK1 blocks in Stage2 and Stage3 are replaced with the proposed L-BTNK1 modules to enhance local facial feature extraction under occlusion. Second, two repeated BTNK2 blocks are removed from Stage4 to reduce the network depth, parameter number, and computational complexity. The other stages are kept consistent with the original ResNet50 to preserve the hierarchical representation ability of the backbone network. For each local facial patch $p_{i}$, LA-ResNet50 is used as a shared feature extractor to obtain its *D*-dimensional feature representation:

(2) $$f_{i}=E(p_{i}),\;f_{i}\in R^{D}$$

where E(⋅) denotes the proposed LA-ResNet50 and *D* denotes the dimension of the extracted feature vector.

**Figure 2:**
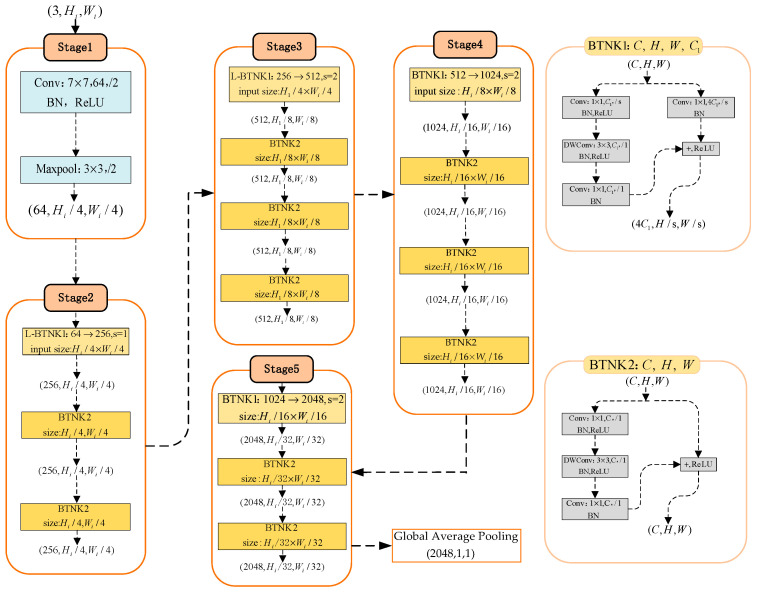
Structure of the proposed LA-ResNet50. The network follows the stage-wise design of ResNet50, where L-BTNK1 is introduced to enhance local feature extraction and part of the repeated BTNK2 blocks are removed to reduce computational complexity. The detailed BTNK1 and BTNK2 structures are shown on the right. Here, S denotes the stride, and *C*_1_ denotes the intermediate channel number in the bottleneck block.

As shown in Figure 2, LA-ResNet50 retains the overall stage-wise architecture of the original ResNet50, including the initial convolutional layer, max-pooling layer, and the subsequent hierarchical feature extraction stages. The input face image first passes through a 7 × 7 convolutional layer, followed by batch normalization, ReLU activation, and max pooling, which reduces the spatial resolution while extracting low-level texture and edge information. The resulting feature maps are then progressively processed by Stage2 to Stage5 to obtain increasingly abstract and discriminative facial representations.

Compared with the original ResNet50, the main modification of LA-ResNet50 lies in the replacement and simplification of bottleneck blocks. Specifically, L-BTNK1 is introduced into Stage2 and Stage3 to enhance local feature representation. Since these stages still retain relatively rich spatial information, introducing L-BTNK1 at this level helps the network capture fine-grained facial details, such as local textures around the eyes, nose, and mouth, which are important for recognizing partially occluded faces. In L-BTNK1, the local pyramid structure combines regular convolution and dilated convolution branches, enabling the network to capture both local details and a larger receptive field without substantially increasing the number of parameters.

In addition, two repeated BTNK2 blocks are removed from Stage4 to achieve lightweight feature extraction. Each BTNK2 block contains multiple convolutional operations, including 1×1 convolution, 3×3 depthwise convolution, and a residual connection. Therefore, removing repeated BTNK2 blocks directly reduces the network depth, the number of convolutional operations, and the overall computational cost. Since Stage4 already contains high-level semantic information, reducing part of the repeated BTNK2 blocks can decrease redundant feature computation while preserving the hierarchical representation ability of the backbone network.

After the hierarchical feature extraction process, the feature maps are further processed by Stage5 and global average pooling to generate the final feature vector. In this way, LA-ResNet50 maintains the multi-stage representation learning capability of ResNet50 while improving local feature enhancement and reducing computational complexity. This design enables the backbone network to extract more robust identity-related features from visible facial regions under partial occlusion conditions.

**Figure 3:**
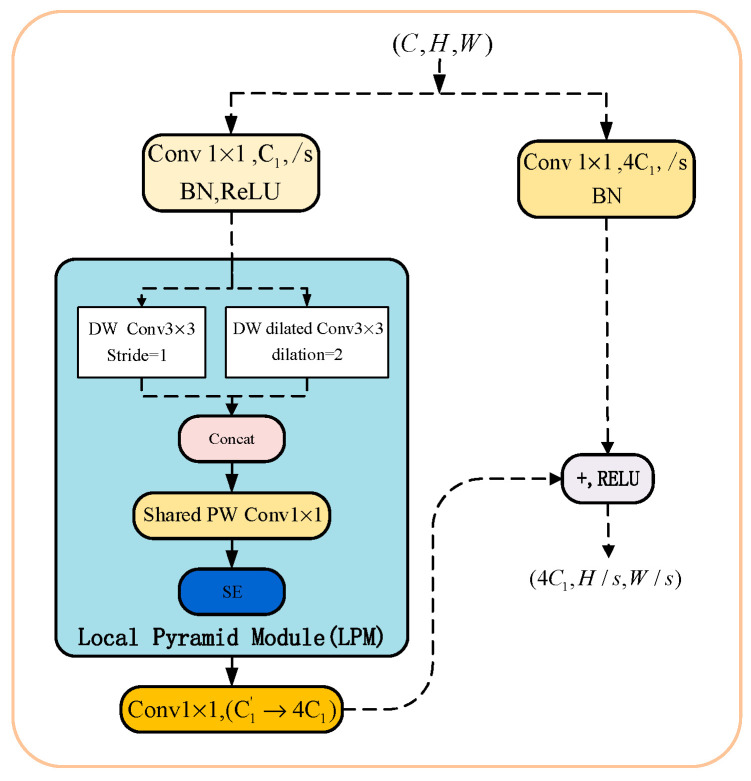
Structure of the proposed L-BTNK1 module. The module adopts a dual-branch local pyramid structure with regular depthwise convolution and dilated depthwise convolution to enhance local feature representation, followed by feature concatenation, shared 1×1 pointwise convolution, SE recalibration, and residual connection.

As shown in Figure 3, the proposed L-BTNK1 module is designed based on the original bottleneck block of ResNet50. Given an input feature map $X\in R^{C\times H\times W}$, 1×1 convolution is first used to reduce the channel dimension. Then, the reduced feature is fed into the Local Pyramid Module (LPM). In the LPM, two parallel convolutionbranches are adopted. One branch uses a 3×3 depthwise convolution to capture fine-grained local facial details, while the other branch uses a 3×3 depthwise dilated convolution [22] with a dilation rate of 2 to enlarge the receptive field and extract contextual information. The outputs of the two branches are concatenated along the channel dimension and then fused by a shared 1×1 pointwise convolution. After multi-scale local feature fusion, a Squeeze-and-Excitation (SE) module is introduced to recalibrate channel-wise feature responses. SE is a classic channel attention module widely used in convolutional neural networks. It adaptively models the importance of different feature channels, thereby enhancing informative channels and suppressing less useful ones. In the proposed L-BTNK1 module, this helps improve the discriminative representation of local facial features while maintaining a lightweight structure. Finally, a 1×1 convolution is used to restore the channel dimension, and the output is combined with the shortcut branch through residual connection and ReLU activation. In this way, L-BTNK1 can enhance local feature representation while maintaining a lightweight structure.

**Figure 4:**
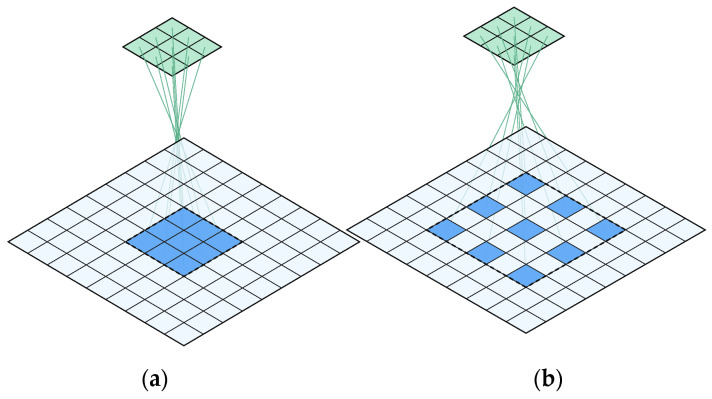
Comparison of receptive fields between regular convolution and standard dilated convolution. (**a**) Regular 3 × 3 convolution. (**b**) Standard 3 × 3 dilated convolution with a dilation rate of 2. The blue grids indicate the sampled positions on the input feature map.

To further illustrate the role of the dilated convolution branch in the Local Pyramid Module (LPM), Figure 4 compares the receptive fields of regular 3 × 3 convolution and dilated 3 × 3 convolution with a dilation rate of 2.

As shown in Figure 4, compared with regular 3×3 convolution, dilated 3×3 convolution with a dilation rate of 2 enlarges the effective receptive field by introducing intervals between adjacent kernel sampling positions. Meanwhile, the number of learnable kernel parameters remains unchanged. Therefore, the dilated convolution branch in the LPM can capture broader contextual information without increasing the number of convolutional parameters, which helps improve local feature representation under partial occlusion.

### 3.4. Learnable Occlusion-Aware Feature Fusion

A lightweight contribution estimation module is designed to assign adaptive reliability weights to different facial patches. Specifically, each local feature vector extracted by LA-ResNet50 is fed into a two-layer MLP with shared parameters to obtain its corresponding original contribution score. Then, Softmax normalization is applied to all patch-level scores to generate reliability weights whose sum equals 1. These weights can adaptively change according to the input features, dynamically reflecting the discriminative value of each local region in the current sample. During training, the module does not rely on explicit occlusion labels, but automatically learns through end-to-end backpropagation with the joint loss. As a result, higher weights are assigned to unoccluded and more discriminative facial patches, while lower weights are given to regions with severe occlusion or noise interference.

Specifically, for the i-th facial patch, its feature representation is denoted as $f_{i}\in R^{D}$. The contribution estimation module adopts a two-layer MLP to map $f_{i}$ to a scalar contribution score. The first fully connected layer performs dimensionality transformation, followed by the ReLU activation function to introduce nonlinear representation capability. Then, the second fully connected layer maps the transformed feature to a scalar value representing the original contribution score of the patch. This process can be expressed as

(3) $$S_{i}=W_{2}\sigma (W_{1}f_{i}+b_{1})+b_{2},$$

where $S_{i}$ denotes the original contribution score of the *i*-th facial patch, $W_{1}$ and $W_{2}$ are learnable weight matrices, $b_{1}$ and $b_{2}$ are bias terms, and σ(⋅) represents the ReLU activation function.

To ensure the relative comparability of contribution scores among different facial patches and constrain the weights to a normalized range, Softmax normalization is applied to the original contribution scores ${\left\{S_{i}\right\}}_{i=1}^{3}$ The final reliability weight of the *i*-th patch is defined as

(4) $$w_{i}=\frac{\exp(S_{i})}{\sum_{j=1}^{3}\exp(S_{j})},$$

where $S_{i}$ denotes the original contribution score of the *i*-th facial patch, and exp(⋅) denotes the exponential function. The resulting weights satisfy $w_{i}\in (0,1)$ and $\sum_{i=1}^{3}w_{i}=1$, and can be directly used for subsequent weighted feature fusion.

The final fused feature representation is obtained by weighted aggregation of the local feature vectors:

(5) $$F_{fused}=\sum_{i=1}^{3}w_{i}f_{i}$$

During training, the contribution estimation module does not rely on any explicit occlusion annotations. Instead, its parameters are learned jointly with the feature extraction network in an end-to-end optimization manner. Since the InfoNCE contrastive learning loss [23] is applied to the fused representation, the gradients can be backpropagated through $F_{fused}$ to the reliability weights $w_{i}$ and the learnable parameters of the MLP, including $\left(W_{1},b_{1},W_{2},b_{2}\right)$. In this way, the module is encouraged to automatically learn reliable weight allocation under different occlusion and interference conditions, thereby achieving occlusion-aware feature fusion and stable feature representation.

### 3.5. Training and Joint Optimization

To enhance both the identity discrimination ability and occlusion robustness of the model, this study constructs a joint optimization objective consisting of ArcFace loss and InfoNCE loss. The joint loss is defined as follows:

(6) $$L_{total}=L_{ArcFace}+\lambda L_{InfoNCE},$$

Here, ArcFace enhances intra-class compactness and inter-class separability by introducing an angular margin constraint, thereby improving identity classification accuracy. However, in locally occluded face recognition scenarios, the reliability of facial features may vary significantly across samples. Severe occlusion can reduce the consistency of identity features and make the angular distribution of samples from the same identity more dispersed. Therefore, directly using a fixed and relatively large angular margin may impose overly strong constraints on low-quality occluded samples and lead to unstable training and noisy gradients [24,25].

For the ground-truth class $y_{i}$, ArcFace replaces the target logit from $\cos\theta_{y_{i}}$ with $\cos(\theta_{y_{i}}+m)$ and introduces a scaling factor (s) to obtain the softmax cross-entropy loss. To address the instability caused by fixed-margin optimization under occlusion, a weight-concentration factor q is introduced, and the angular margin is reformulated as m(q), resulting in the adaptive ArcFace loss:

(7) $$L_{ArcFace}=-\frac{1}{N}\sum_{i=1}^{N}\log(\frac{\exp(s\cdot \cos(\theta_{y_{i}}+m(q)))}{\exp(s\cdot \cos(\theta_{y_{i}}+m(q)))+\sum_{j\neq y_{i}}\exp(s\cdot \cos\theta_{j})})$$

The factor q is derived from the local-region reliability weights $w_{i=1}^{K}$, where $\sum_{i=1}^{K}w_{i}=1,\;w_{i}\in (0,1)$. In this study, q is defined as the sum of squared local-region reliability weights:

(8) $$q=\sum_{i=1}^{K}w_{i}^{2}$$

According to q, a linear mapping is used to construct the adaptive angular margin:

(9) $$m(q)=m_{\max}-(m_{\max}-m_{\min})q,\;0<m_{\min}<m_{\max},$$

When q is large, the regional weight distribution is more concentrated, indicating that only a few facial regions are reliable and that the sample may suffer from severe occlusion. In this case, a smaller angular margin m(q) is assigned to avoid imposing overly strong constraints on low-quality occluded samples and to reduce the influence of unstable gradients on class centers and decision boundaries.

Conversely, when q is small, the regional weights are more evenly distributed, indicating that multiple facial regions are reliable and sufficient identity-related information is preserved. In this case, a larger angular margin m(q) can be assigned to enhance inter-class separability and improve discriminative ability.

This study adopts an InfoNCE-based contrastive loss to introduce identity-guided supervision. InfoNCE is a commonly used contrastive learning objective that constructs positive and negative samples in the feature space. In this study, samples belonging to the same identity are treated as positive samples, while samples from different identities are treated as negative samples. By increasing the similarity between positive samples and reducing the similarity between the anchor and negative samples, the model can learn feature representations with stronger discriminative ability and stability.

For a given fused feature $F_{fused}$ used as an anchor, the InfoNCE loss is defined as follows:

(10) $$L_{\mathrm{InfoNCE}}=-\log\frac{\exp(\mathrm{sim}(F_{fused},F^{+})/\tau )}{\exp(\mathrm{sim}(F_{fused},F^{+})/\tau )+\sum_{K=1}^{K}\exp(\mathrm{sim}(F_{fused},F_{k}^{-})/\tau )},$$

where $F_{k}^{+}$ denotes the positive sample feature belonging to the same identity as the anchor, and $F_{k}^{-}$ represents the negative sample feature from a different identity. The function sim(⋅,⋅) denotes the cosine similarity between two features, and τ is the temperature factor used to adjust the smoothness of the similarity distribution.

For the InfoNCE loss, positive and negative samples are constructed according to identity labels. For a given anchor sample, images with the same identity are treated as positive samples, even if they contain different occlusion types or occluded regions. In contrast, images from different identities are treated as negative samples regardless of their occlusion patterns. This sampling strategy encourages the model to maintain intra-identity feature consistency under different occlusion conditions and improves inter-identity separability in the feature space.

The joint loss acts on both LA-ResNet50 and the MLP-based contribution estimation module during backpropagation, enabling collaborative optimization of feature extraction and reliability weight learning. Specifically, ArcFace strengthens the overall identity discrimination boundary, while InfoNCE constrains feature consistency across different occlusion conditions. Through this joint optimization, the MLP-based module can learn to assign higher weights to unobstructed and more discriminative regions, while assigning lower weights to regions affected by severe occlusion or noise interference. As a result, the proposed method achieves more robust local feature fusion and identity representation.

### 3.6. Similarity Calculation and Recognition

After local feature extraction and adaptive feature fusion, the final fused feature representation is obtained for the input face image. This fused feature is used for subsequent face matching and identity recognition.

Similarity measurement plays a central role in the face matching process. Following common face embedding-based recognition protocols [26], cosine similarity is calculated between the fused feature of the input image and the feature vectors stored in the face database. Cosine similarity measures the angular closeness between two feature vectors and is defined as follows:

(11) $$sim(F_{1},F_{2})=\frac{F_{1}\cdot F_{2}}{{\left\|F_{1}\right\|}_{2}{\left\|F_{2}\right\|}_{2}}$$

where $F_{1}$ and $F_{2}$ denote two feature vectors, $F_{1}\cdot F_{2}$ represents their dot product, and ${\left\|\cdot \right\|}_{2}$ denotes the L2 norm. The value of cosine similarity ranges from −1 to 1, and a larger value indicates a higher similarity between the two feature vectors.

In this study, the similarity threshold was set to 0.78 based on the statistical distribution of same-identity similarity scores on the validation set. During recognition, the fused feature of the input image is compared with all feature vectors in the face database, and the similarity scores are sorted in descending order. If the highest similarity score exceeds the predefined threshold, the identity label corresponding to the most similar database feature is returned as the recognition result.

## 4. Experiments and Results

### 4.1. Experimental Setup

In all experiments, the models were trained using the AdamW optimizer for 300 epochs, with a batch size of 32 and an initial learning rate of 0.05. During training, a stepwise learning rate decay strategy was adopted to dynamically adjust the learning rate. All experiments were conducted on a workstation with the hardware and software configurations detailed in Table 1.

For the loss function settings, following commonly used settings in margin-based face recognition and contrastive learning, several candidate values of s, m, and τ were evaluated on the validation set. The final selected settings are summarized in Table 2.

Among the tested settings, s=64, m=0.5, and τ=0.07 achieved stable convergence and satisfactory recognition performance under occlusion conditions. Therefore, these values were adopted as the final hyperparameter settings in our experiments.

LFW was selected as the basic dataset for the experiment [27]. After quality screening, a total of 3360 facial images from 1680 identities were retained.

All facial images were first aligned and uniformly cropped to an input resolution of 224 × 224. For occluded samples, the corresponding occluded versions were synthesized following the method in [28]. The final Ma_LFW dataset was constructed, containing 1680 identities and 13,600 images. The dataset was divided into training, validation, and test sets at a ratio of 7:1:2. Specifically, the training set contained 1176 identities and 9520 images, the validation set contained 168 identities and 1360 images, and the test set contained 336 identities and 2720 images.

For face verification evaluation, 6000 test pairs were constructed following the standard LFW protocol, including 3000 positive pairs from the same identity and 3000 negative pairs from different identities.The detailed distribution of different occlusion types across the training, validation, and test sets of the Ma_LFW dataset is summarized in Table 3.

**Figure 5:**
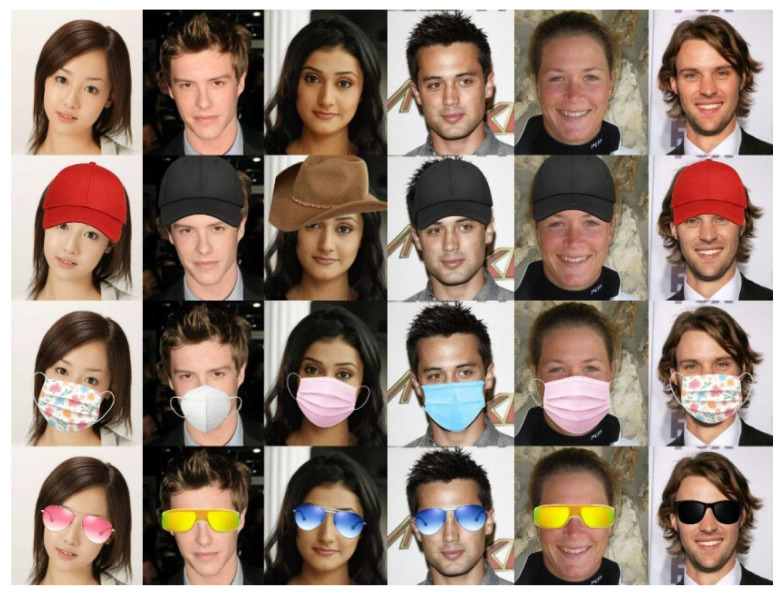
Examples of original and synthetically occluded face images in the Ma_LFW dataset. The first row shows original face images, while the following rows show representative samples with hat, mask, and sunglasses occlusions. These samples illustrate the construction of occluded face images used for local region generation and feature extraction.

To evaluate the robustness of our proposed method under various occlusion conditions, we constructed a synthetic occlusion dataset based on Ma_LFW, as illustrated in Figure 5. The figure presents representative samples of original and synthetically occluded face images, including hats, masks, and sunglasses, which serve as the input for subsequent local region generation and feature extraction.

To simulate possible interference during practical image acquisition, Gaussian noise and salt-and-pepper noise were additionally introduced into the occluded test samples as test perturbations. Gaussian noise was used to simulate continuous random interference caused by sensor noise or low-light imaging conditions, whereas salt-and-pepper noise was used to simulate impulse interference that may occur during image acquisition or transmission. It should be noted that these noise perturbations were only used to construct more challenging test samples and were not involved in model training.

**Figure 6:**
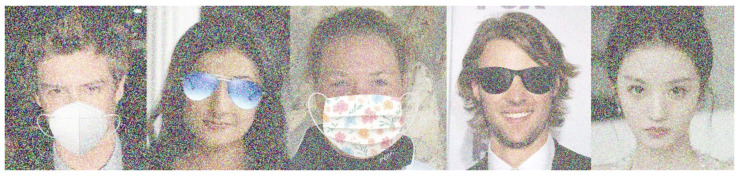
Representative occluded face images with Gaussian noise.

**Figure 7:**
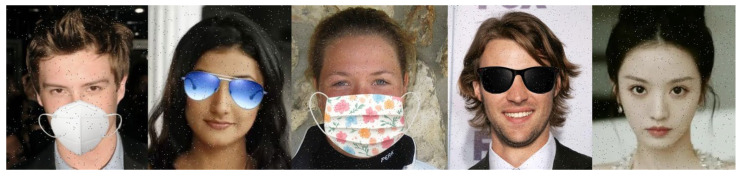
Representative occluded face images with salt-and-pepper noise.

Figure 6 and Figure 7 show representative occluded face images with Gaussian noise and salt-and-pepper noise, respectively.

**Figure 8:**
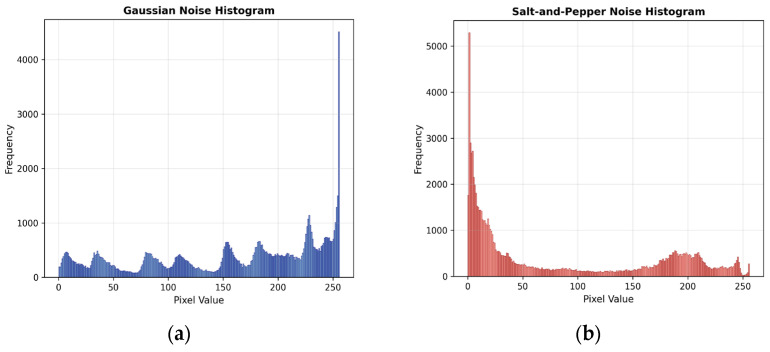
Pixel histograms of occluded face images under different noise perturbations: (**a**) Gaussian noise and (**b**) salt-and-pepper noise.

The pixel histograms in Figure 8 further illustrate the different distribution characteristics of the two noise types. Gaussian noise causes continuous variations in pixel values, whereas salt-and-pepper noise causes some pixels to concentrate near the extreme values of 0 and 255.

It should be noted that these noise perturbations were used only as additional interference conditions in the test samples to construct a more challenging testing environment and were not involved in model training. They were not treated as independent experimental variables for separate quantitative analysis. Therefore, the main quantitative evaluation of this study focuses on occlusion robustness, including different occlusion types, occlusion ratios, ablation studies, and comparisons with existing methods.

### 4.2. Evaluation Metrics

The confusion matrix is the core tool for evaluating model performance, and we use it to record the test results of each model. The specific design is shown in Table 4. Among them, TP (True Positive) represents that the model judges the sample as a positive class, and the sample actually belongs to the positive class; FN (False Negative) refers to the model predicting a negative class, but the true class of the sample is positive; FP (False Positive) indicates that the model judges the class as positive, but the actual class is negative; TN (True Negative) means that the model predicts a negative class, and the true class of the sample is also negative [29].

Selecting accuracy, the False Acceptance Rate (FAR), and the False Rejection Rate (FRR) as performance evaluation indicators for the recognition model, these indicators can reflect the recognition performance of the recognition model from different perspectives. The evaluation of model performance should not only focus on the recognition results themselves, but also need to be comprehensively analyzed in conjunction with actual deployment requirements.

Accuracy is used to measure the proportion of correctly classified results in the model output, which can be understood as the ratio of correct results to the total sample size.

(12) $$\mathrm{Accuracy}=\frac{TP+TN}{TP+FP+TN+FN}$$

The FAR is the ratio of misidentifying different people’s faces as the same person, measuring the security of the model.

(13) $$\mathrm{FAR}=\frac{FP}{FP+TN}$$

The FRR is the ratio of misjudging the same person’s face as different people, measuring the usability of the model.

(14) $$\mathrm{FRR}=\frac{FN}{FN+TP}$$

In addition, Params and FPS were used to evaluate the model complexity and inference efficiency. Params denotes the number of model parameters and reflects the storage cost of the model. FPS denotes the number of images processed per second during testing and reflects the real-time inference capability of the model. In general, fewer parameters and higher FPS indicate better suitability for practical deployment.

### 4.3. Ablation Study

To verify the effectiveness of each component in the proposed framework, seven ablation models are designed, as shown in Table 5. Model 1 is used as the baseline model with the original ResNet50 backbone and ArcFace loss. Models 2 and 3 are built on ResNet50 by introducing region-aware fusion and InfoNCE, respectively, to evaluate their individual effects. Model 4 replaces ResNet50 with LA-ResNet50 to evaluate the lightweight backbone. Based on LA-ResNet50, Model 5 introduces region-aware fusion without InfoNCE, while Model 6 introduces InfoNCE without region-aware fusion. Finally, Model 7 combines LA-ResNet50, region-aware fusion, and InfoNCE, forming the complete proposed model. Since ArcFace is used as the basic classification loss in all settings, the InfoNCE column indicates whether the additional contrastive loss is introduced.

As shown in Table 5, Model 7 achieves the best overall performance among all ablation models, with the highest accuracy of 92.3%. Compared with Model 1, Model 4 replaces the original ResNet50 backbone with LA-ResNet50, improving the accuracy from 82.4% to 88.9% while reducing the number of parameters from 25.7 M to 21.1 M. This result indicates that the proposed LA-ResNet50 backbone can improve recognition performance while reducing model complexity.

Models 2 and 3 are used to separately evaluate the individual effects of region-aware fusion and InfoNCE based on the original ResNet50 backbone. Compared with Model 1, Model 2 improves the accuracy from 82.4% to 89.5%, indicating that region-aware fusion helps enhance the feature representation of reliable local facial regions. Model 3 achieves an accuracy of 90.3%, showing that InfoNCE can improve identity feature consistency under occlusion conditions.

To further analyze the effects of region-aware fusion and InfoNCE under the same LA-ResNet50 backbone, Models 5 and Models 6 are additionally introduced. Compared with Model 4, Model 5 improves the accuracy from 88.9% to 89.7%, indicating that region-aware fusion can still bring performance improvement on the LA-ResNet50 backbone. Model 6 achieves an accuracy of 91.2%, suggesting that InfoNCE also improves model performance under the same lightweight backbone.

More importantly, the comparison between Model 5 and Model 7 directly demonstrates the contribution of InfoNCE when the LA-ResNet50 backbone and region-aware fusion are kept unchanged. After introducing InfoNCE, the accuracy increases from 89.7% to 92.3%. Similarly, compared with Model 6, Model 7 further improves the accuracy from 91.2% to 92.3%, verifying the complementary effect between region-aware fusion and InfoNCE. Although Model 7 has a slightly larger number of parameters than Model 4 and Model 6, it still has fewer parameters than the original ResNet50-based models while achieving the best recognition accuracy and the FRR. These results verify the effectiveness of each component and demonstrate that the complete framework achieves a better balance between recognition performance and model complexity.

To further verify whether the performance differences between the complete model and each ablation model are statistically significant, McNemar’s test was con-ducted on the same test set [30]. Since all models were evaluated using the same testing samples, McNemar’s test is suitable for paired classification results. For each comparison, $n_{01}$ denotes the number of samples incorrectly classified by the ablation model but correctly classified by the complete model, while $n_{10}$ denotes the number of samples correctly classified by the ablation model but incorrectly classified by the complete model. The test statistic is calculated as follows:

(15) $$\chi^{2}=\frac{{\left(|n_{01}-n_{10}|-1\right)}^{2}}{n_{01}+n_{10}}.$$

A result with (*p* < 0.05) is considered statistically significant. As shown in Table 6, the *p*-values between the complete model and all ablation models are lower than 0.05, indicating that the improvements achieved by the complete model are statistically significant. These results further demonstrate the effectiveness of the proposed adaptive local feature fusion strategy and joint loss optimization.

As shown in Table 6, all comparisons between Model 5 and the ablation models achieve statistically significant differences, with all *p*-values lower than 0.05. This indicates that the performance improvements of the complete model are not caused by random variation in the test samples, but are statistically reliable.

### 4.4. Comparative Experiments

To verify the effectiveness of the improved method proposed in this paper, ArcFace, CurricularFace, and AdaFace were selected as comparative models for experiments. These methods are representative margin-based face recognition methods. To ensure fairness and comparability, all methods were trained and tested under the same experimental environment and evaluation protocol. The overall performance comparison is shown in Table 7.

As shown in Table 7, the proposed method achieves the best overall performance among all compared methods. Specifically, our method obtains an accuracy of 92.3%, which is higher than ArcFace, CurricularFace, and AdaFace by 9.9%, 6.5%, and 4.1%, respectively. In terms of error rates, the proposed method achieves the lowest FAR of 6.5% and the lowest FRR of 8.9%, indicating better security and usability in face verification under occlusion conditions.

In addition, the proposed method has 22.2 M parameters, which is lower than ArcFace, CurricularFace, and AdaFace. This demonstrates that the proposed LA-ResNet50 can reduce model complexity while maintaining strong feature representation ability. Meanwhile, our method achieves the highest FPS of 114, indicating better inference efficiency. These results show that the proposed framework achieves a better balance between recognition accuracy, error rate, model complexity, and inference speed.

The performance improvement can be mainly attributed to two aspects. First, LA-ResNet50 introduces local pyramid bottleneck modules and removes redundant bottleneck blocks, which enhances local multi-scale feature extraction while reducing unnecessary computation. Second, the region-aware feature fusion strategy and the joint optimization with ArcFace and InfoNCE enable the model to emphasize reliable visible facial regions and maintain identity feature consistency under different occlusion conditions. Therefore, the proposed method achieves more robust recognition performance under partial occlusion.

### 4.5. Robustness Analysis

#### 4.5.1. Sensitivity Analysis on Occlusion Ratio

**Figure 9:**
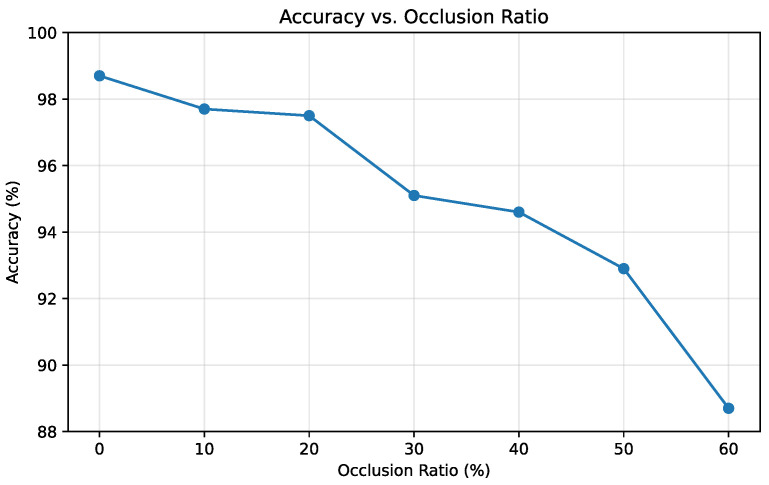
Sensitivity analysis of recognition accuracy under different occlusion ratios.

To further evaluate the robustness of the proposed method under different occlusion severities, a sensitivity analysis to occlusion ratio was conducted. Based on a subset of 672 images selected from the original test set, controlled occlusion-ratio test subsets were constructed. The occlusion ratio was defined as the ratio of the occluded facial area to the total facial area. In this experiment, the occlusion ratios were set to 0%, 10%, 20%, 30%, 40%, 50%, and 60%. Each occlusion-ratio subset contained 672 test images, and the same evaluation protocol was applied to all subsets. The accuracy under different occlusion ratios is shown in Figure 9. Each point in the figure represents the recognition accuracy obtained at a specific occlusion ratio, and the connected line is used to illustrate the overall performance trend as the occlusion ratio increases.

As shown in Figure 9, when the occlusion ratio is between 0% and 20%, a relatively large amount of identity-related facial information is still preserved. Therefore, even if some local regions are occluded, the model can still rely on other visible regions for identity discrimination, resulting in only a slight decrease in accuracy.

When the occlusion ratio increases to 30–50%, some key identity-related regions begin to be affected, and the completeness of local features is reduced. Although the region-aware fusion mechanism can alleviate the influence of occluded regions to some extent, the available identity information becomes more limited, leading to a decline in recognition accuracy.

When the occlusion ratio reaches 60%, more than half of the facial area is occluded, and the occlusion block is likely to cover multiple key facial regions simultaneously. In this case, the available visible identity-discriminative information is substantially reduced, and both local feature extraction and regional reliability estimation are affected. Although the model can suppress the feature weights of occluded regions, it becomes difficult to extract sufficient stable identity features from the remaining visible regions. As a result, a more pronounced decrease in accuracy is observed.

#### 4.5.2. Comparison Under Different Occlusion Types

To further evaluate the robustness of the proposed method under different occlusion types, experiments were conducted on four occlusion subsets, including mask, hat, sunglasses, and random occlusion. These occlusion types correspond to different facial regions and interference patterns. Mask occlusion mainly affects the lower facial region, hat occlusion mainly affects the upper facial region, sunglasses occlusion mainly covers the eye region, and random occlusion may appear at different facial positions. All methods were evaluated under the same Ma_LFW test protocol, and the recognition accuracy under each occlusion type is reported in Table 8.

As shown in Table 8, the proposed method achieves the highest recognition accuracy under all four occlusion types. Specifically, our method obtains accuracies of 93.3%, 94.5%, 89.8%, and 90.8% under mask, hat, sunglasses, and random occlusion conditions, respectively. These results indicate that the proposed local-region reliability estimation and adaptive feature fusion strategy can effectively reduce the influence of occluded regions and enhance the representation of reliable visible facial regions.

Among the four occlusion types, sunglasses and random occlusions cause relatively greater performance degradation for all methods, because they may cover key identity-related regions or introduce irregular interference. Nevertheless, the proposed method still maintains the best performance under these challenging conditions. This further demonstrates the robustness and adaptability of the proposed framework under different occlusion patterns.

#### 4.5.3. Model Attention Visualization Analysis Under Occlusion

**Figure 10:**
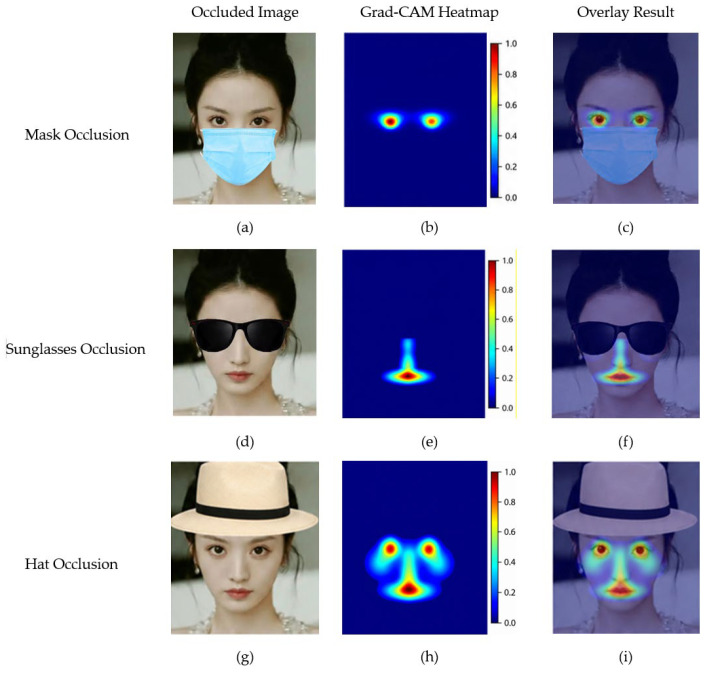
Grad-CAM attention visualization results under different occlusion conditions. The first column shows the occluded input images, the second column shows the Grad-CAM heatmaps, and the third column shows the overlay results. (**a**–**c**) Occluded images (Mask, Sunglasses, Scarf); (**d**–**f**) Corresponding heatmaps; (**g**–**i**) Corresponding overlay results.

To further examine whether the proposed method can focus on reliable and discriminative facial regions under occlusion, a model attention visualization analysis was conducted under different occlusion conditions. The purpose of this analysis is to provide an intuitive understanding of how the model responds to visible and occluded facial regions, thereby further validating the effectiveness of the proposed occlusion-aware feature fusion strategy. Specifically, representative face images with mask, sunglasses, and hat occlusions were selected for visualization. Grad-CAM was then applied to generate the corresponding attention heatmaps, which reflect the spatial distribution of model responses. In Figure 10, the first column shows the occluded input images, the second column presents the Grad-CAM attention heatmaps, and the third column shows the overlay results obtained by superimposing the heatmaps on the corresponding occluded images.

In the attention heatmaps, different colors represent different levels of model response intensity. Red and yellow regions indicate high-response areas, suggesting that these regions are more strongly associated with the model’s recognition decision. Green regions represent moderate responses, whereas blue regions correspond to low-response areas, including background regions or less informative regions, indicating that these areas have relatively limited influence on the recognition decision. As shown in Figure 10, the attention distribution changes with the occlusion type because the reliable visible facial regions differ under different occlusion conditions. When the lower facial region is occluded by a mask, the model mainly focuses on the visible eye region. When the eye region is occluded by sunglasses, the attention shifts toward the nose and mouth regions. In the case of hat occlusion, where the upper facial region is partially occluded, the model assigns higher responses to visible regions such as the eyes, nose, and mouth. These visualization results demonstrate that the proposed method can adaptively adjust its attention according to the occlusion location, emphasizing visible and informative facial regions while reducing the influence of occluded or less reliable regions.

## 5. Discussion

Although the proposed method achieves improved robustness under several common occlusion conditions, it still has some limitations that need to be further discussed. In particular, the current framework mainly focuses on frontal or near-frontal face images with partial occlusion, while its performance may be affected when severe pose variations and occlusions occur simultaneously. Under extreme pose variations, especially when the yaw angle of the face exceeds 45°, some key facial regions may become invisible or severely distorted. In this case, the fixed horizontal three-region partition may no longer accurately correspond to the semantic upper, middle, and lower facial regions. For example, the eye region, nose bridge, or mouth region may suffer from spatial displacement, compression, or overlap with occlusions due to head rotation, which may reduce the accuracy of local feature extraction and regional reliability estimation. When large pose variations and severe occlusions occur simultaneously, the amount of visible identity-related information is further reduced. As a result, the model may have difficulty assigning reliable region-level fusion weights, leading to unstable identity feature representation.

To alleviate this problem, future work can introduce pose-aware face alignment or 3D face normalization methods to transform large-yaw face images into a more unified frontal representation space. In addition, adaptive region generation strategies based on facial landmark detection or attention mechanisms can be explored, allowing local regions to be dynamically adjusted according to pose variations and occlusion locations rather than relying on a fixed horizontal partition. Furthermore, multi-pose data augmentation and pose-invariant feature learning can be incorporated to enhance the model’s ability to represent identity features under profile views, occlusions, and their combined conditions.

In practical deployment, privacy and ethical issues related to face recognition should also be considered. Since facial images contain sensitive biometric information, data collection and use should follow relevant privacy regulations and ethical guidelines, including user consent, secure storage, and prevention of unauthorized data disclosure.

## 6. Conclusions

This paper proposes an occlusion-aware face recognition method to address the degradation of recognition performance caused by missing local facial information and occlusion noise under partial occlusion. The proposed method adopts ResNet50 as the basic feature extraction backbone and improves it into a lightweight LA-ResNet50 network by introducing local pyramid bottleneck blocks. Through multi-scale feature modeling within local regions, the network can capture fine-grained texture information and local structural cues at different scales. This design enhances the extraction of discriminative features from visible facial regions, such as the eye region, nose bridge, and forehead. Meanwhile, redundant computations are reduced while maintaining feature representation capability, thereby improving feature learning efficiency and reducing computational complexity.

In the feature fusion stage, this paper further designs an identity-guided occlusion-aware feature fusion strategy. Specifically, the face image is divided into three horizontal facial regions, and the reliability of each region is predicted by an MLP-based module. Softmax normalization is then used to generate adaptive fusion weights, allowing the model to emphasize effective identity information from unoccluded or weakly occluded regions while suppressing the influence of heavily occluded regions on the overall representation. In addition, contrastive learning constraints are introduced to encourage features of the same identity under different occlusion conditions to maintain higher consistency in the feature space, while improving the separability between different identities. This further enhances the discriminative ability and robustness of the model.

The experimental results demonstrate that the designed modules contribute positively to recognition performance under different occlusion conditions. The performance improvement can be attributed to two main factors. First, local multi-scale feature enhancement strengthens the representation of visible facial regions. Second, reliability modeling and adaptive weighted fusion reduce the interference of occlusion noise on identity features, thereby achieving more accurate and stable face recognition in complex occlusion scenarios. These results verify the feasibility and effectiveness of combining local feature enhancement with reliability-weighted fusion for occluded face recognition.

In future work, lightweight deployment optimization for mobile and edge devices will also be further considered. On the one hand, structured pruning can be used to remove redundant channels or convolution kernels, thereby reducing the number of parameters and computational cost. On the other hand, knowledge distillation can be adopted to transfer discriminative knowledge from a larger teacher model to a lightweight student model, so as to maintain recognition accuracy while reducing model complexity. In addition, re-parameterization techniques can be combined to preserve multi-branch structures during training and equivalently convert them into simpler single-branch structures during inference, thereby improving inference speed and deployment efficiency. Further optimization strategies, such as TensorRT acceleration and quantization-aware training, can also be considered for practical deployment on resource-constrained devices.

## Figures and Tables

**Table 1:** Hardware and software configurations used in the experiments.

Category	Configuration
CPU	Intel(R) Core(TM) i7-14650HX 2.20 GHz
GPU	NVIDIA GeForce RTX 4060 with 8 GB memory
Operating System	Windows 11
Deep Learning Environment	Python 3.8; PyTorch 2.1; CUDA 11.8;
Image Processing Libraries	OpenCV 4.8; NumPy 1.24

**Table 2:** Candidate values and selected hyperparameter settings.

Parameter	Candidate Values	Selected Value
s	32, 48, 64, 80	64
m	0.3, 0.4, 0.5, 0.6	0.5
τ	0.03, 0.05, 0.07, 0.10	0.07

**Table 3:** Distribution of different occlusion types in the training, validation, and test sets of the Ma_LFW dataset.

Occlusion Type	Training Set	Validation Set	Test Set	Total
Mask	2856	408	816	4080
Hat	2856	408	816	4080
Sunglasses	2856	408	816	4080
Random	952	136	272	1360
Total	9520	1360	2720	13,600

**Table 4:** Confusion matrix used for performance evaluation. Rows represent true labels, and columns represent predicted labels.

	Predict	Positive	Negative
Real			
Positive		TP	FN
Negative		FP	TN

**Table 5:** Ablation study of different components in the proposed framework.

	Backbone	Region-Aware Fusion	InfoNCE	Accuracy	Params
Model 1	ResNet50	✕	✕	82.4%	25.7 M
Model 2	ResNet50	✓	✕	89.5%	25.7 M
Model 3	ResNet50	✕	✓	90.3%	25.8 M
Model 4	LA-ResNet50	✕	✕	88.9%	21.1 M
Model 5	LA-ResNet50	✓	✕	90.7%	22.2 M
Model 6	LA-ResNet50	✕	✓	91.2%	21.1 M
Model 7	LA-ResNet50	✓	✓	92.3%	22.2 M

“✓” denotes that the corresponding module is incorporated into the model, and “✕” denotes that it is not.Model 1 is the baseline model using ResNet50 and ArcFace loss. Models 2 and 3 introduce region-aware fusion and InfoNCE based on ResNet50, respectively. Model 4 replaces ResNet50 with LA-ResNet50. Models 5 and 6 introduce region-aware fusion and InfoNCE based on LA-ResNet50, respectively. Model 7 combines LA-ResNet50, region-aware fusion, and InfoNCE as the complete model.

**Table 6:** McNemar’s test results between the complete model and ablation models.

Comparison	$n_{01}$	$n_{10}$	$\chi^{2}$	*p*-Value
Model 5 vs. Model 1	644	50	508.41	<0.05
Model 5 vs. Model 2	239	35	151.88	<0.05
Model 5 vs. Model 3	223	55	101.53	<0.05
Model 5 vs. Model 4	165	45	68.57	<0.05

**Table 7:** Overall performance comparison with representative face recognition methods on the Ma_LFW test set.

	Accuracy	FAR	FRR	Params	FPS
ArcFace [31]	82.4%	12.3%	11.1%	25.7 M	110
CurricularFace [32]	85.8%	11.4%	15.4%	25.7 M	109
AdaFace [33]	88.2%	10.2%	10.2%	25.8 M	111
Ours	92.3%	6.5%	8.9%	22.2 M	114

**Table 8:** Recognition accuracy of different methods under different occlusion types.

	Mask	Hat	Sunglasses	Random
ArcFace [31]	88.9%	87.3%	81.4%	78.6%
CurricularFace [32]	89.3%	92.1%	85.4%	83.4%
AdaFace [33]	90.2%	93.5%	86.7%	86.0%
Ours	93.3%	94.5%	89.8%	90.8%

## Data Availability

The data supporting the findings of this study are available from the authors upon reasonable request.

## Abbreviations

The following abbreviations are used in this manuscript:

GAN	Generative Adversarial Network
MLP	Multi-Layer Perceptron
InfoNCE	Information Noise-Contrastive Estimation
FAR	False Acceptance Rate
FRR	False Rejection Rate
FPS	Frames Per Second
